# Exploiting the dynamic properties of covalent modification cycle for the design of synthetic analog biomolecular circuitry

**DOI:** 10.1186/s13036-016-0036-1

**Published:** 2016-11-14

**Authors:** Mathias Foo, Rucha Sawlekar, Declan G. Bates

**Affiliations:** Warwick Integrative Synthetic Biology Centre, School of Engineering, University of Warwick, Coventry, CV4 7AL UK

**Keywords:** Covalent modification cycle, Chemical reaction networks, Analog synthetic biomolecular circuits, Linear and nonlinear operators, Feedback control systems, Synthetic biology applications

## Abstract

**Background:**

Cycles of covalent modification are ubiquitous motifs in cellular signalling. Although such signalling cycles are implemented via a highly concise set of chemical reactions, they have been shown to be capable of producing multiple distinct input-output mapping behaviours – ultrasensitive, hyperbolic, signal-transducing and threshold-hyperbolic.

**Results:**

In this paper, we show how the set of chemical reactions underlying covalent modification cycles can be exploited for the design of synthetic analog biomolecular circuitry. We show that biomolecular circuits based on the dynamics of covalent modification cycles allow (a) the computation of nonlinear operators using far fewer chemical reactions than purely abstract designs based on chemical reaction network theory, and (b) the design of nonlinear feedback controllers with strong performance and robustness properties.

**Conclusions:**

Our designs provide a more efficient route for translation of complex circuits and systems from chemical reactions to DNA strand displacement-based chemistry, thus facilitating their experimental implementation in future Synthetic Biology applications.

## Background

The emerging field of Synthetic Biology [1, 2] has provided a number of recent examples of the successful construction of digital circuits in cells, including biomolecular transistors [3, 4] and logic gates [5–7]. A major open challenge associated with such digital circuitry is to ensure robust functionality of the designed circuit in the presence of interactions with the host cell [8, 9]. One possible solution to this problem is to develop analog circuitry, [10], since in general analog designs require far fewer devices to carry out a given computation at the moderate precision needed in cells, resulting in lower resource requirements and a reduced metabolic burden on the cell [11, 12]. There is also a growing realisation that in many cases the use of purely digital designs can fail to exploit the hybrid approach to information processing used by cells, which often combines digital computation with analog processing of signals with different levels of gradation [13]. The ability to process signals with different levels of gradation will be a key requirement for the development of more complex synthetic circuits with advanced monitoring and control capabilities.

Transduction of external perturbations via signalling cascades is a classical examples of analog signal processing in the cell, [14–16]. One of the most ubiquitous motifs seen in cell signalling cascades is the cycle of covalent modification [17, 18], examples of which include phosphorylation/dephosphorylation of enzymes [19], DNA methylation [20] and monoclonal antibodies [21]. The covalent modification cycle is implemented via a highly concise set of chemical reactions, which have been shown in [17, 22] to potentially exhibit highly sigmoidal input-output characteristics, generating so-called ultrasensitive or hyperbolic responses (see also [23, 24]). In [25], the authors systematically examine the steady-state and dynamic responses of covalent modification cycles to time varying perturbations and demonstrate the existence of two additional types of regimes, termed signal-transducing and threshold-hyperbolic, giving a total of four distinct mapping regimes (see Figure 2 of [25]).

Thus, by modifying only the reaction rates for the chemical reactions governing the covalent modification cycle, four highly distinct input-output mapping behaviours can be obtained. Here, we show how this flexibility can be exploited for the design of synthetic analog biomolecular circuitry. From an engineering design point of view, this versatile input-output mapping property is highly attractive, as different combinations of these input-output mappings can be used to design circuits that can perform many different types of computation, information processing, and control. From an experimental implementation point of view, the extremely concise set of chemical reactions used in covalent modification is also very advantageous, since it facilitates circuit designs with fewer reactions and components, hence easing their implementation using DNA-based chemistry.

## Methods

### Chemical reactions underlying the covalent modification cycle motif

Whereas signals in systems and control theory can have both positive and negative values, this is not the case for biomolecular concentration, as they can only take non-negative values. This issue can be addressed following the framework suggested in [26]. Here, we present a summarised version of this framework, for full details the reader is referred to [26].

A signal *x* is represented as the difference in concentration of two chemical species, *x* ^+^ and *x* ^−^. Thus, *x* ^+^ and *x* ^−^ are, respectively, the positive and negative components of *x* such that *x*=*x* ^+^−*x* ^−^. From an implementation point of view, e.g. using DNA-based chemistry as proposed in [27], *x* ^+^ and *x* ^−^ can each represent a DNA strand and the final concentration of *x* can be recovered using *x*=*x* ^+^−*x* ^−^. As an example, consider an initial DNA strand, *x* ^+^ with 10nM concentration present in the system. Then, another DNA strand, *x* ^−^ with 20nM concentration is added to the system and the resulting signal *x* becomes negative. In terms of the underlying chemical reactions, ensuring proper implementation of *x*=*x* ^+^−*x* ^−^, requires a fast annihilation reaction between *x* ^+^ and *x* ^−^, i.e. $x^{+} + x^{-} \xrightarrow {\eta } \emptyset $ with *η* is the annihilation rate.

In this work, we adopt this formalism as it enables the realisation of negative signals. While there are alternative formalism available from standard chemical reaction network theory (see e.g. [28]) these cannot deal with negative signals or realise two-sided subtraction operators, which are required, for example in feedback control design to compute the difference between two signals, where the resulting difference can result in either a positive or negative signal.

**Fig. 1:**
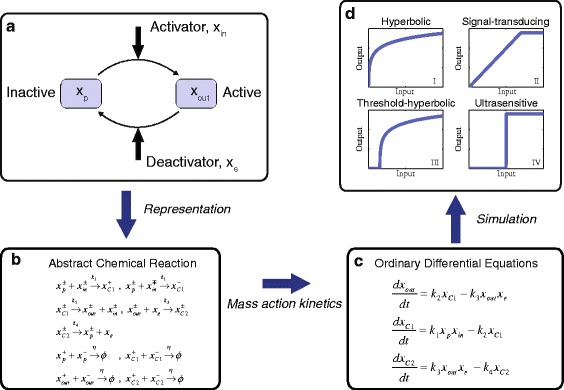
The dynamics of the covalent modification cycle. **a** The schematic of the covalent modification cycle. **b** The abstract chemical reaction representation of the covalent modification cycle. **c** The abstract chemical reaction can be described by ordinary differential equations (ODE’s) using mass action kinetics. **d** Depending on the choice of reaction rates, the set of ODE’s exhibits four distinct mapping regimes [25]: (I) Hyperbolic. (II) Signal-transducing. (III) Threshold-hyperbolic. (IV) Ultrasensitive

For conciseness, we use the notation *x* ^±^→*x* ^±^+*y* ^∓^ to represent a pair of reactions, *x* ^+^→*x* ^+^+*y* ^−^ and *x* ^−^→*x* ^−^+*y* ^+^. In [26] and [27] it was shown how sets of abstract chemical reactions implemented using this formalism can be used to design biomolecular circuits that compute a number of linear operators, such as scalar gains, summation/subtraction and integration. Circuits designed using this framework can then be implemented experimentally using DNA-based chemistry, as discussed in [29–31]. Using the mathematical formalism of [26], we can represent a covalent modification cycle of an enzyme-substrate pair by the following set of 14 abstract chemical reactions:

1 $$ {{}\begin{aligned} x_{p}^{\pm} &+ x_{in}^{\pm} \xrightarrow{k_{1}} x_{C1}^{+}, \hspace{5pt} x_{p}^{\pm} + x_{in}^{\mp} \xrightarrow{k_{1}} x_{C1}^{-}, \hspace{5pt} x_{C1}^{\pm} \xrightarrow{k_{2}} x_{out}^{\pm} + x_{in}^{\pm} \\ x_{out}^{\pm} &+ x_{e} \xrightarrow{k_{3}} x_{C2}^{\pm}, \hspace{5pt} x_{C2}^{\pm} \xrightarrow{k_{4}} x_{p}^{\pm} + x_{e}, \hspace{5pt} x_{p}^{+} + x_{p}^{-} \xrightarrow{\eta} \emptyset\\ x_{C1}^{+} &+ x_{C1}^{-} \xrightarrow{\eta} \emptyset, \hspace{5pt} x_{out}^{+} + x_{out}^{-} \xrightarrow{\eta} \emptyset, \hspace{6pt} x_{C2}^{+} + x_{C2}^{-} \xrightarrow{\eta} \emptyset \\ \end{aligned}}  $$

where all the *x* represent biomolecular species and *k*_*j*_ (*j*=1,⋯,4) are the reaction rates. The covalent modification cycle described by Eq. (1) is illustrated in Fig. 1(a), and operates in the following manner. $x_{p}^{\pm }$ represents the inactive component, which is associated with $x_{in}^{\pm }$, (i.e., the enzyme kinase), forming an intermediate species, $x_{C1}^{\pm }$ with reaction rate *k*_1_. The intermediate species then produces the active component, $x_{out}^{\pm }$ and the remaining unused $x_{in}^{\pm }$ with reaction rate *k*_2_. This reaction from inactive state to active state is called the forward reaction. Likewise, the active component, $x_{out}^{\pm }$ is associated with *x*_*e*_ (i.e. the enzyme phosphatase) with reaction rate *k*_3_ and produces another intermediate species, $x_{C2}^{\pm }$. This intermediate species produces inactive component, $x_{p}^{\pm }$ and the remaining unused *x*_*e*_ with reaction rate *k*_4_. This reaction from active state to inactive state is called the backward reaction. Note that as *x*_*e*_ is externally introduced, it is not split into positive and negative components.

Using mass action kinetics, (see e.g. [32, 33]), the nonlinear ordinary differential equations (ODE’s) for the covalent modification cycle are given by:

2 $$\begin{array}{*{20}l}  \frac{dx_{out}}{dt} &= k_{2}x_{C1} - k_{3}x_{out}x_{e}  \\ \frac{dx_{C1}}{dt} &= k_{1}x_{p}x_{in} - k_{2}x_{C1} \\ \frac{dx_{C2}}{dt} &= k_{3}x_{out}x_{e} - k_{4}x_{C2} \end{array} $$

with total concentration *x*_*total*_=*x*_*e*_+*x*_*C*2_ is constant and these ODE’s produce four distinct mapping regimes for the different choices of reaction rates, [25] (see Fig. 1(d)).

### Implementation of chemical reactions via DNA-based chemistry

A number of recent studies have described how synthetic circuits composed of abstract chemical reactions may be readily implemented in DNA-based chemistry (see e.g. [29, 30, 34, 35]). As highlighted in [35], these chemical reactions can serve as a programming language for designing synthetic circuits based on DNA-based chemistry. Mathematically expressed components of circuits designed using DNA can be derived from biologically synthesised plasmids, in principle enabling the in vitro implementation of those circuits. A particular advantage of employing DNA-based chemistry lies in the ease of implementation, given that the design relies on the choice of relevant sequences following the well-known Watson-Crick (i.e. adenine-thymine and guanine-cytosine) pairing. In [30], it is shown that unimolecular and bimolecular chemical reactions can be compiled into DNA strand displacement (DSD)-based chemistry to achieve the desired behaviour of the considered biomolecular circuit. Here, we present a summarised version of the framework and refer interested readers to [30] for more details.

A simple bimolecular DSD reaction can be described by the following reaction,



where *δ*_*b*_ and *δ*_*ub*_ are the binding and unbinding rate of the DNA strand respectively. This reaction begins when *Q*, called the invader strand, binds with *X* in a complementary manner at the toe-hold domain of *X*. When this binding takes place, parts of the strand of *X* are displaced and this disengagement results in product *Y* and waste, *R*. The partially double stranded product, *Y* can then bind with other toe-hold domains of other DNA complexes. Usually, the toe-hold domain has short nucleotides (6-10 nucleotides) to ensure the reaction is fast and reliable. By varying the value of *δ*_*ub*_ and *δ*_*b*_, one can control the rate of reaction. Specifically, the binding and unbinding rates can be changed by changing the length of the DNA strands. To further elaborate, the reaction rates from chemical reactions can be mapped into these binding and unbinding rates of the DNA reactions following the framework of [30]. Based on these binding and unbinding rates, the designer can determine the length of DNA strands required to achieve that reaction.

Given that different DNA strands do not interact directly with each other, their interaction is normally mediated by auxiliary species that are usually present in large amounts. The framework of approximating abstract chemical reactions to DSD presented in [30] considers the DNA implementation for unimolecular and bimolecular reactions, where these two types of reactions can be represented by Eqs. (4) and (??) respectively.

4 $$ X_{1} + G \xrightarrow{q} O~\text{and}~O + T \xrightarrow{q_{max}} X_{2} + X_{3}  $$

where *G*, *O*, *T*, *L*, *H*, and *B* are auxiliary species with appropriate initial concentrations *C*_*max*_, *q*=*δ*/*C*_*max*_ is the partial strand displacement rate and *q*_*max*_ is the maximum strand displacement rate.

The set of chemical reactions governing the covalent modification cycle is made up exclusively of unimolecular and bimolecular reactions, and thus, following the framework shown above, all of the circuits described in the remainder of this paper can be implemented via nucleic acids. Note, however, that the experimental challenges associated with building such circuitry increase with the number of reactions that are required for a given circuit. It is therefore imperative that circuit designs utilise as few reactions as possible, in order to ease experimental implementation and maximise scalability of the resulting synthetic systems.

## Results and discussions

### Computing nonlinear operators

Here, we present analog biomolecular circuit designs, based on the chemical reactions underlying the covalent modification cycle, that can compute three important nonlinear operators – the logarithm of arbitrary base, the signum function and the absolute value of a signal. We show that these designs achieve a dramatic reduction in circuit complexity when compared with designs based on purely abstract chemical reaction networks.

#### Computing a logarithm of arbitrary base

Consider the operation *c*=log_*b*_ *a*, i.e. computing the logarithm of *a* to the base *b*. This logarithm can be computed through the change of logarithm base, i.e. $c = \frac {\ln a}{\ln b}$, where ln denotes the natural logarithm. In other words, *c* can be realised as a ratio of ln*a* and ln*b*. Several numerical methods exist to compute the natural logarithm. The most commonly used method is to use Taylor series, but this method accurately computes the logarithm of numbers only within the range 0<*x*<2. A more efficient method to compute the natural logarithm for *x*≥2 is based on the area hyperbolic tangent series approximation [36]. Thus, using such approximation, the natural logarithm can be computed as follows:

6 $$  \ln(x) = \ln\left(\frac{z-1}{z+1}\right) = 2\sum_{i=0}^{l} \frac{z^{2i+1}}{2i+1}  $$

where *l* is the order of the series. The larger the order *l* is, the better the approximation, but the higher the complexity of the circuit. In this paper, we choose *l*=10 as this order allows us to compute the logarithm of numbers up to 10.

**Fig. 2:**
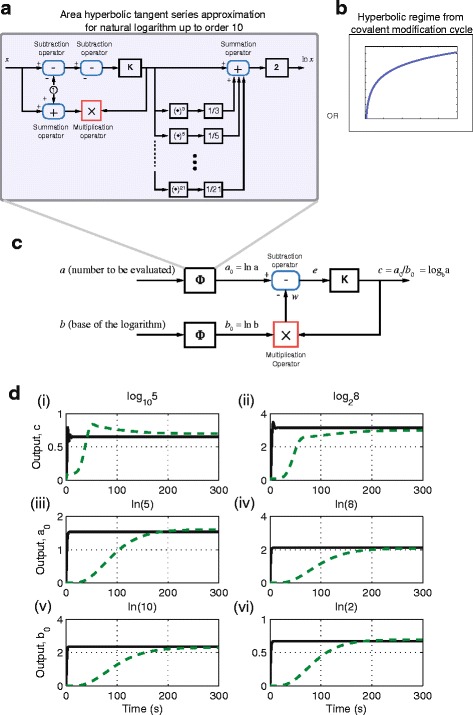
Computation of logarithm of arbitrary base. **a** Block diagram of a circuit to compute natural logarithm based on the area hyperbolic tangent series approximation of order *l*=10. **b** The hyperbolic regime obtained from covalent modification cycle. **c** Block diagram of a circuit to compute logarithm of arbitrary base by taking the ratio of two natural logarithms. **d** Simulation results for (i) log105. (ii) log28. (iii) ln5. (iv) ln8. (v) ln10. (vi) ln2. In all simulations, *Green* dashed-dot line: using area hyperbolic tangent series approximation up to order 10. *Black* solid line: using hyperbolic regime from the covalent modification cycle

The block diagram of a circuit that can compute the natural logarithm using the area hyperbolic tangent series approximation of order *l*=10 is shown in Fig. 2(a). This circuit uses a combination of several linear and nonlinear operators; summation, subtraction, gain, multiplication and power exponent, each of which may be implemented using a number of abstract chemical reactions. For details of the chemical reactions used in computing the linear summation, subtraction and gain operators, see [26], (the numbers of reactions needed are 7, 7, and 5 respectively). For the nonlinear multiplication and power exponent operators, their abstract chemical reactions (modified from [28] and [37]) are given as follows.

*Multiplication:* Consider the following multiplication: *y*=*u*_1_ *u*_2_. At steady-state, the set of abstract chemical reactions that realise this operator is given by $u_{1}^{\pm } + u_{2}^{\pm } \xrightarrow {\gamma _{M}} u_{1}^{\pm } + u_{2}^{\pm } + y^{+}$, $u_{1}^{\pm } + u_{2}^{\mp } \xrightarrow {\gamma _{M}} u_{1}^{\pm } + u_{2}^{\mp } + y^{-}$, $y^{\pm } \xrightarrow {\gamma _{M}} \emptyset $ and $y^{+} + y^{-} \xrightarrow {\eta } \emptyset $, where *γ*_*M*_ is the multiplication reaction rate. In total, 7 abstract chemical reactions are required to realise the multiplication operator, whose ODE is given by $\frac {dy}{dt} = \gamma _{M}(u_{1}u_{2} - y)$.

*Power exponent:* Consider the following exponent: *y*=*u* ^*n*^, where *n* is a positive integer. At steady-state, the set of abstract chemical reactions that realise this operator is given by

$$\begin{array}{*{20}l} u^{\pm} &+ u^{\pm} \xrightarrow{\gamma_{P}} u^{\pm} + u^{\pm} + (u^{2})^{+}\\ u^{\pm} &+ u^{\mp} \xrightarrow{\gamma_{P}} u^{\pm} + u^{\mp} + (u^{2})^{-}\\ (u^{2})^{\pm} &\xrightarrow{\gamma_{P}} \emptyset\\ (u^{2})^{+} &+ (u^{2})^{-} \xrightarrow{\eta} \emptyset\\ & \vdots \notag \\ u^{\pm} &+ (u^{n-1})^{\pm} \xrightarrow{\gamma_{P}} u^{\pm} + (u^{n-1})^{\pm} + y^{+}\\ u^{\pm} &+ (u^{n-1})^{\mp} \xrightarrow{\gamma_{P}} u^{\pm} + (u^{n-1})^{\mp} + y^{-}\\ y^{\pm} &\xrightarrow{\gamma_{P}} \emptyset\\ y^{+} &+ y^{-} \xrightarrow{\eta} \emptyset \end{array} $$

where *γ*_*P*_ is the power exponent reaction rate. In total, 7(*n*−1) abstract chemical reactions are required to realise the power exponent operator, and the corresponding ODE is given by $\frac {dy}{dt} = \gamma _{P}\Big \{\Big (\prod _{l=1}^{n} u\Big) - y\Big \}$.

With *l*=10, we require 13 summation and subtraction operators, one multiplication operator, 10 power exponent operators with exponents 3,5,…,21 and 12 gain operators. This results in a total of $13(7) + 1(7) + \sum _{n=3,5,\ldots,21} 7(n-1) + 12(5) = 928$ abstract chemical reactions. To compute the logarithm of arbitrary base, as shown in Fig. 2(c), we require one more *Φ* that computes the second natural logarithm and one each for the subtraction, multiplication and gain operator. Thus, this circuit requires a total of 2(928)+1(7)+1(7)+1(5)=1875 abstract chemical reactions, which makes it completely intractable from an experimental point of view.

The huge number of abstract chemical reactions required to implement the circuit described above prompted us to seek alternative, more efficient, designs. We note that the characteristic of a natural logarithm resembles the hyperbolic regime of the covalent modification cycle (see Fig. 1(d)-i) thus, making this regime potentially useful for computing the natural logarithm. Interestingly, this response is not governed by the order of the series approximation. Thus, as long as one can obtain the appropriate reaction rates for *k*_1_ to *k*_4_, we can compute the natural logarithm. Moreover, this approach requires only 14 abstract chemical reactions. To compute the logarithm of arbitrary base using this approach, we replace the *Φ* block in Fig. 2(c) with the covalent modification cycle reactions in the hyperbolic regime, as shown in Fig. 2(b). This results in a total of 2(14) + 7 + 7 + 5 = 47 abstract chemical reactions, a 97 % reduction in circuit complexity.

Simulation results for computing log105 and log28 are shown in Fig. 2(D-i) and (D-ii) respectively. To implement the hyperbolic response, reaction rates are *k*_1_=0.22 /M/s, *k*_2_=0.43 /s, *k*_3_=1.03 /M/s, *k*_4_=35.10 /s, and *x*_*e*_=1 M.

For both approaches, the computed logarithms are close to the actual value, however the circuit based on the covalent modification cycle motif is significantly faster in settling to the correct steady-state value, even though it uses far fewer chemical reactions. An alternative approach for the biological computation of logarithms has been designed and implemented in [13]. This approach utilises transcriptional regulation, which requires a host cell, while our approach can be implemented in cell-free conditions (e.g. using DNA Strand Displacement (DSD) framework). Moreover, [13] considers the computation of the natural logarithm, while our approach enables the computation of logarithms of arbitrary base.

#### Computing the signum function

**Fig. 3:**
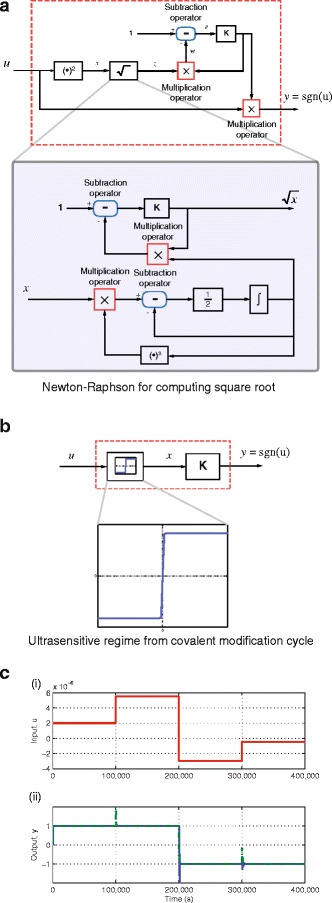
Computing the signum function. **a** Block diagram of a circuit to compute the signum function. In computing the square root, the Newtown-Raphson method is used. **b** Block diagram of a circuit to compute the signum function using the ultrasensitive regime from the covalent modification cycle. **c** Computation of signum function (i) Input signal. (ii) Output of the signum function. *Green* dashed-dot line: using combination of operators. *Blue* dashed line: using ultrasensitive regime from covalent modification cycle

A general signum function outputs +1 for any positive real valued input and -1 for any negative real valued input. Mathematically, this is represented as *X*=sgn(*X*)|*X*|. The signum function can be designed using the circuit shown in Fig. 3(a). Details of the three abstract chemical reactions used to realise the integrator used in this circuit are provided in [26]. In this circuit, the input signal is first squared before taking its square root. The square root operator can be implemented using the well-known Newton-Raphson method, which requires two subtraction operators, two multiplication operators, one power exponent operator of order 3, one integrator operator and one gain operator, resulting in a total of 2(7) + 2(7) + 1(14) + 1(3) + 1(5) = 50 reactions. After taking the square root, the reciprocal of this signal is calculated, and this is then multiplied by the input signal, which results in either +1 or -1 depending on the sign of the input signal. With a power exponent operator of order 2, one square root operator, one subtraction operator, one gain operator and two multiplication operators, the total number of reactions required to compute the signum function is 1(7) + 1(50) + 1(7) + 1(5) + 2(7) = 83.

Now, the characteristics of a signum function closely resemble the ultrasensitive regime of the covalent modification cycle. Thus, as shown in Fig. 3(b), the corresponding chemical reactions can also be utilised to compute the signum function. While the ultrasensitive regime alone could be used to compute the signum function, we go a step further by appending a gain operator, *K* that can be used for scaling purposes. With the introduction of the gain operator, the total number of reactions required to compute the signum function is now only 14 + 1(5) = 19 reactions, a reduction in complexity of 77 %.

In the simulation results shown in Fig. 3(c), the input to our signum function is in the range of 10^−6^ while the output is in the range of 10^−3^. We could have specified the signum function to produce outputs in the range of ±1 instead of ±1×10^−3^, however, there could arise situations where such a response is not achievable due to the physical constraints on the system. In view of this, the gain component can be used to scale the output from ±10^−3^ to ±1. This ultrasensitive response is obtained with *k*_1_=5,000 /M/s, *k*_2_=5 /s, *k*_3_=50 /M/s, *k*_4_=0.05 /s and *x*_*e*_=0.1*μ* M.

Fig. 3(c) shows the simulation results of the signum function computed using both circuits. The input is changed every 10,000 seconds starting with 2.0×10^−6^, 5.5×10^−6^, −3.0×10^−6^ and −0.5×10^−6^. The simulation result shows that while both circuits are able to compute the signum function accurately, the much simpler circuit based on covalent modification exhibits smaller transient overshoots in response to changes in the value of the input signal.

#### Computing the absolute value of a signal

**Fig. 4:**
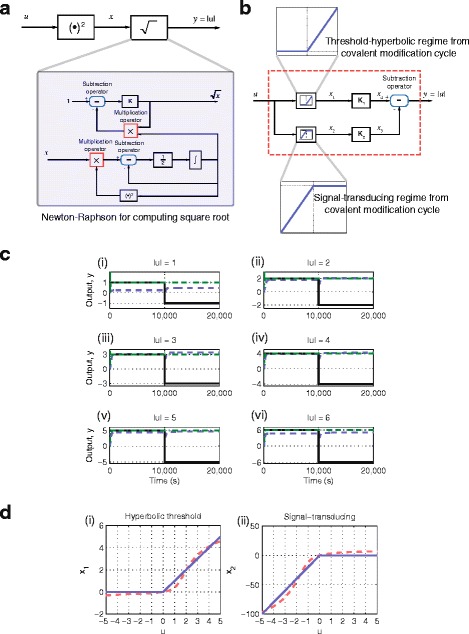
Computing the absolute value. **a** Block diagram of a circuit to compute the absolute value using a combination of operators. **b** Block diagram of a circuit to compute the absolute value using the threshold-hyperbolic and signal-transducing regimes from the covalent modification cycle. **c** Computation of absolute value. *Black solid line*: the input value. *Green dashed-dot line*: using combination of operators. *Blue dashed line*: using threshold-hyperbolic and signal-transducing regimes from the covalent modification cycle. (i) Input, *u*=±1 M, (ii) Input, *u*=±2 M. (iii) Input, *u*=±3 M. (iv) Input, *u*=±4 M. (v) Input, *u*=±5 M. (vi) Input, *u*=±6 M. **d** Comparison between ideal (*Blue solid line*) and simulated (*Red dashed line*) responses. (i) Threshold-hyperbolic response. (ii) Signal-transducing response

The block diagram of a circuit that can compute the absolute value of a signal is shown in Fig. 4(a). As shown, the input signal is first squared before taking its square root. We have introduced the Newton-Raphson method previously for computing the square root and thus it can be seen that this circuit requires a total of 7 + 50 = 57 reactions.

We shall now illustrate how the remaining two regimes of the covalent modification cycle, i.e. signal-transducing and threshold-hyperbolic, can be utilised to compute the absolute value of a signal, for which the block diagram is shown in Fig. 4(b). The threshold-hyperbolic regime has a dead-zone, (i.e. a non-responsive region given any input signal) followed by a hyperbolic-like region. To compute the absolute value, the dead-zone range must not respond to input signals, *u* that are strictly negative and then respond to input signals that are non-negative in a linear manner. To achieve this, note that in the threshold-hyperbolic regime, any hyperbolic response has an “almost linear” region when the input signal is small. By taking advantage of this property, we can ensure that our required threshold-hyperbolic regime has a linear instead of hyperbolic response after the dead-zone region. On the other hand, the signal-transducing regime has a linear region followed by a plateau region. This makes this regime suitable for responding only to non-positive input signals and not to strictly positive input signals. We also introduce two gain components, *K*_1_ and *K*_2_ for scaling purposes. By combining these two regimes with two gain components and one subtraction operation, 2(14) + 2(5) + 7 = 45 reactions are required to compute the absolute value, a reduction in circuit complexity of 21 %.

Since the threshold-hyperbolic response has a linear response with unity gradient, there is no requirement for the gain block, *K*_1_ or equivalently, *K*_1_=1. To achieve this threshold-hyperbolic response, we set *k*_1_=0.0027 /M/s, *k*_2_=16,640 /s, *k*_3_=0.043 /M/s, *k*_4_=0.008 /s and *x*_*e*_=3.5 M. For the signal-transducing response, suppose that due to the limitations imposed by the system, a unity gradient of the linear response cannot be achieve, resulting in the gradient of the linear response to be 20. In this case, the gain component is set to *K*_2_=1/20. To achieve this signal-transducing response, we set *k*_1_=5 /M/s, *k*_2_=100 /s, *k*_3_=5 /M/s, *k*_4_=630 /s and *x*_*e*_=1.8 M. Fig. 4(c) shows the simulation results for six different input signals, *u* ranging from +1 M to +6 M. At time 10,000 s, these input signals, *u* are switched to their negative counterpart ranging from -1 M to -6 M.

From Fig. 4(c), we see that the circuit using the configuration shown in Fig. 4(a) performs very well. For the circuit using the covalent modification cycle, the performance is also excellent, although when *u*=±1 M and ±6 M some deviations are observed. This is because the threshold-hyperbolic and signal-transducing responses achieved are not a perfect match to the ideal responses, as shown in Fig. 4(d).

#### Remarks on choosing the reaction rates of the covalent modification cycle

In all our illustrations above, the reaction rates (i.e. *k*_1_ to *k*_4_) of the covalent modification cycle are obtained via numerical optimisation using the MATLAB ^*Ⓡ*^ function ‘*fminsearch*’. The numerical optimisation aims to find the reaction rates (within biologically valid ranges) that minimise the difference between the desired mapping regime of the covalent modification cycle and the one obtained from Eq. (2).

### Designing nonlinear controllers

Here, we illustrate how the chemical reactions underlying the covalent modification cycle can be used for the design of nonlinear biomolecular feedback controllers.

#### Controller descriptions

**Fig. 5:**
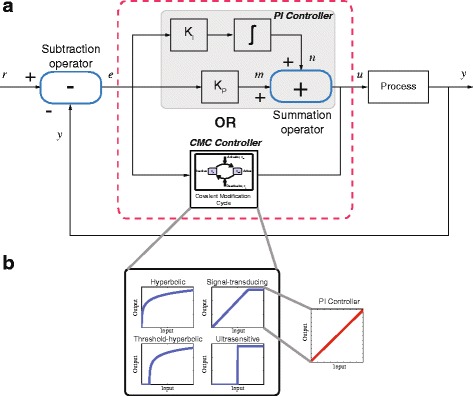
**a** Block diagram configuration of a biomolecular feedback control system. **b** Four different mappings of input-output signals in a covalent modification cycle [25]. The signal-transducing mapping resembles the steady-state input-output mapping of a PI controller

Figure 5(a) shows the block diagram of a biomolecular feedback control circuit. In industrial control systems, the most commonly used controller is the linear proportional-integral (PI) controller, and this type of controller has been successfully implemented for biomolecular systems using DNA-based chemistry in previous studies [26, 27]. Interestingly, the signal-transducing regime of the covalent modification cycle resembles the steady-state input-output mapping of the PI controller (Fig. 5(b)). Here, we compare the performance and robustness properties of a nonlinear ‘covalent modification cycle’ (CMC) controller, designed to operate in its signal-transducing regime, with those of a classical PI controller.

The modules involved in the biomolecular feedback controllers are as follows.

*PI controller:* The classical PI controller considered here is designed according to the methodology of [26]. The PI controller is made up of one integrator, one proportional gain and one summation operator. These three sub-modules require a total of 15 abstract chemical reactions to implement as follows:

*Integrator:* $e^{\pm } \xrightarrow {K_{I}} e^{\pm } + n^{\pm }$ and $n^{+} + n^{-} \xrightarrow {\eta } \emptyset $, where *K*_*I*_ is the integral gain of the PI controller and *η* is the annihilation rate.

*Proportional gain:* $e^{\pm } \xrightarrow {\gamma _{K}K_{P}} e^{\pm } + m^{\pm }$, $m^{\pm } \xrightarrow {\gamma _{K}} \emptyset $ and $m^{+} + m^{-} \xrightarrow {\eta } \emptyset $, where *K*_*P*_ is the proportional gain of the PI controller, *γ*_*K*_ is the gain reaction rate.

*Summation junction:* $m^{\pm } \xrightarrow {\gamma _{Sm}} m^{\pm } + u^{\pm }$, $n^{\pm } \xrightarrow {\gamma _{Sm}} n^{\pm } + u^{\pm }$, $u^{\pm } \xrightarrow {\gamma _{Sm}} \emptyset $, and $u^{+} + u^{-} \xrightarrow {\eta } \emptyset $, where *γ*_*Sm*_ is the summation reaction rate.

The tuning of this PI controller involves adjusting *K*_*P*_, *K*_*I*_ and the reaction rates *γ*_*K*_ and *γ*_*Sm*_.

*CMC controller:* The chemical reactions required to implement the CMC controller are given by Eq. (1) with *e*=*x*_*in*_ and *u*=*x*_*out*_. We choose values for the CMC controller’s reaction rates that place it in its signal-transducing input-output mapping regime, which closely resembles the steady-state input-output mapping of a PI Controller (see Fig. 5(b)). Note that the CMC controller requires 14 reactions to implement, 1 fewer than the PI controller.

#### Closed-loop system and ODE approximations

Comparative performance of the two controllers is evaluated for two biomolecular processes - the first a simple first order linear process and the second a more complex second order nonlinear process. The abstract chemical reactions for both processes are given by

*Linear process:* $u^{\pm } \xrightarrow {k_{p1}} u^{\pm } + y^{\pm }$, $y^{\pm } \xrightarrow {k_{p2}} \emptyset $ and $y^{+} + y^{-} \xrightarrow {\eta } \emptyset $, where *k*_*p*1_ and *k*_*p*2_ are the catalysis and degradation rates of the process.

*Nonlinear process:* $u^{\pm } + p^{\pm } \xrightarrow {k_{r1}} q^{+}$, $u^{\pm } + p^{\mp } \xrightarrow {k_{r1}} q^{-}$, $q^{\pm } \xrightarrow {k_{r2}} y^{\pm } + p^{\pm }$, $y^{\pm } \xrightarrow {k_{r3}} \emptyset $ and $y^{+} + y^{-} \xrightarrow {\eta } \emptyset $, where *p* and *q* are intermediate species involved in the second order process reaction. *k*_*r*1_, *k*_*r*2_ and *k*_*r*3_ are respectively the binding, catalytic and degradation rates of the process. For the subtraction operator, the abstract chemical reactions are ${r}^{\pm } \xrightarrow {\gamma _{Sb}} {r}^{\pm } + {e}^{\pm }, {y}^{\pm } \xrightarrow {\gamma _{Sb}} {y}^{\pm } + {e}^{\pm }, {e}^{\pm } \xrightarrow {\gamma _{Sb}} \emptyset $ and ${e}^{+} + {e}^{-} \xrightarrow {\eta } \emptyset $.

Using generalised mass action kinetics, the ODEs corresponding to the abstract chemical reactions employed in the modules of the feedback control system are given as:

*Subtraction operator*:

7 $$  \frac{de}{dt} = \gamma_{Sb}(r - y - e)  $$

*PI controller*:

8 $$\begin{array}{*{20}l}  \frac{dn}{dt} &= K_{I}e  \\ \frac{dm}{dt} &= \gamma_{K}(K_{P}e - m) \\ \frac{du}{dt} &= \gamma_{Sm}(m + n - u) \end{array} $$

*CMC controller*:

9 $$\begin{array}{*{20}l}  \frac{du}{dt} & = k_{2}x_{C1} - k_{3}ux_{e} \\ \frac{dx_{C1}}{dt} &= k_{1}e - k_{2}x_{C1} \\ \frac{dx_{C2}}{dt} & = k_{3}ux_{e} - k_{4}x_{C2} \end{array} $$

*Linear process*:

10 $$ \frac{dy}{dt} = k_{p1}u - k_{p2}y  $$

*Nonlinear process*:

11 $$\begin{array}{*{20}l}  \frac{dq}{dt} &= k_{r1}up - k_{r2}q  \\ \frac{dy}{dt} & = k_{r2}q - k_{r3}y \end{array} $$

According to the formalism of [26], the gain and summation operators used in the PI controller require multiple identical reaction rates to be used in their sets of abstract chemical reactions. However, implementing this requirement in an experimental setting is unlikely to be feasible, as experimental biologists are rarely able to specify the reaction rates of chemical reactions exactly. Additionally, in practice, as highlighted in [26], unregulated chemical devices or leaky expressions could potentially affect production and degradation rates and subsequently alter the behaviour of the designed component. To investigate these issues, we perform a formal robustness analysis of both controllers, focussed on the effect of uncertainties in the implemented reaction rates on the closed-loop stability and performance properties of the feedback system.

#### Performance analysis of controllers with linear process

To analyse the performance and robustness of the closed-loop responses achieved by the feedback controllers with the linear process, step response tests and Monte Carlo simulations are performed, respectively. For the Monte Carlo simulations, all the parameters are randomly drawn from a uniform distribution. The number of Monte Carlo simulations required to achieve various levels of estimation uncertainty with known probability are calculated using the well-known Chernoff bound [38]. Following the guidelines provided in [39], an accuracy level of 0.05 and a confidence level of 99 % are chosen for the Monte Carlo simulation analysis, which requires a total number of 1060 simulations [38, 40]. To investigate the effect of different levels of uncertainty we vary the parameters within ranges of 20 %, 50 %, 100 % and 120 % around their nominal values. Mathematically, we have *p*(1+*Δ* *P*(*x*)), where *p*∈{*γ*_*Sb*1_,*γ*_*Sb*2_,*γ*_*Sb*3_,*γ*_*K*1_,*γ*_*K*2_,*γ*_*Sm*1_,*γ*_*Sm*2_,*γ*_*Sm*3_,*K*_*I*_,*K*_*P*_,*k*_1_,*k*_2_,*k*_3_, *k*_4_,*k*_*p*1_,*k*_*p*2_}, *P*(*x*) is the probability distribution and *Δ*∈{0.2,0.5,1.0,1.2}. Note that we split reaction rates *γ*_*Sb*_, *γ*_*K*_ and *γ*_*Sm*_ according to the number of chemical reactions in which they are involved.

In our simulations, a step change in the concentration of the reference species, *r* from 0 M to 1 M occurs at time 0 s and the purpose of the controller is to ensure that the process output reaches this new desired concentration. As quantitative measures of the control system performance, the step response characteristics, which comprise rise time, *t*_*r*_, settling time, *t*_*s*_, percentage of overshoot, *M*_*OV*_ and steady-state error, *e*_*ss*_ are used [41]. For good closed-loop performance, it is desirable to achieve a small *t*_*r*_, *t*_*s*_ and *M*_*OV*_ as well as having *e*_*ss*_=0. As a benchmark for comparison, we first calculate the step response characteristics without parameter uncertainty. Hereafter, we refer to these as the set of results for the *nominal system*. The parameters for the nominal system in the required abstract chemical reactions are:

*Linear process:* *k*_*p*1_=0.1 /s, *k*_*p*2_=0.1 /s.

*PI controller:* *γ*_*Sb*1_, *γ*_*Sb*2_, *γ*_*Sb*3_=0.4 /s, *γ*_*Sm*1_, *γ*_*Sm*2_, *γ*_*Sm*3_=0.8 /s, *γ*_*K*1_, *γ*_*K*2_=0.0004 /s, *K*_*P*_=1 and *K*_*I*_=0.045.

*CMC controller:* *k*_1_, *k*_3_=0.00185 /M/s, *k*_2_, *k*_4_=0.5 /s. *x*_*p*_+*u*+*x*_*C*1_+*x*_*C*2_=27.5 M and *x*_*e*_+*x*_*C*2_=0.033 M.

*Subtractor dynamics:* *γ*_*Sb*1_, *γ*_*Sb*2_, *γ*_*Sb*3_=0.4 /s.

**Fig. 6:**
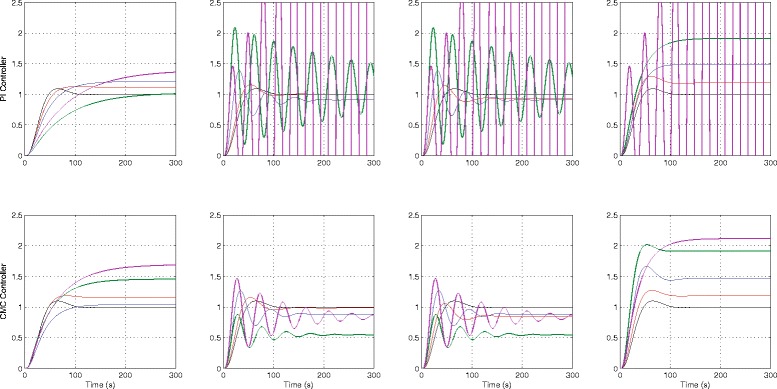
*Top row*: PI controller + linear process. *Bottom row*: CMC controller + linear process. Nominal and worst cases of *t*_*r*_, *t*_*s*_, *M*_*OV*_ and *e*_*ss*_ with *Δ*=0.2,0.5,1.0,1.2. *Black* line: Nominal system. *Red* line: worst-case response for *Δ*=0.2, *Blue* line: worst-case response for *Δ* = 0.5, *Green* line: worst-case response for *Δ* = 1.0, *Magenta* line: worst-case response for *Δ* = 1.2

The step response characteristics for both the nominal systems are tabulated in Table 1. For each of the analysed uncertainty sets, the worst-case values returned by Monte Carlo simulation for each of the step response characteristics and its associated parameter set are shown. Note that a range of parameters is given here as the parameter set associated with each worst-case characteristic is different. For example, the parameters yielding the worst *t*_*r*_ may not yield the worst *t*_*s*_, *M*_*OV*_ and *e*_*ss*_ and vice versa. For illustration, the step responses depicting the nominal and worst-case responses for each step response characteristics for *Δ*∈{0.2,0.5,1.0,1.2} are shown in Fig. 6 for both PI and CMC controllers.

**Table 1:** Step response characteristics and worst-case parameter ranges for PI and CMC controllers + linear process

PI controller					
Characteristics	Nominal	*Δ*=0.2	*Δ*=0.5	*Δ*=1.0	*Δ*=1.2
*t*_*r*_ (s)	29	44	75	157	173
*t*_*s*_ (s)	96	113	175	499	Unstable
*M*_*OV*_ (%)	9.14	22.83	52.25	114.17	Unstable
*e*_*ss*_ (M)	0.00	0.19	0.49	0.91	Unstable
Parameters	Nominal	*Δ*=0.2	*Δ*=0.5	*Δ*=1.0	*Δ*=1.2
*γ*_*Sb*1_ (/s)	0.400	0.431–0.476	0.532–0.595	0.475–0.791	0.584–0.599
*γ*_*Sb*2_ (/s)	0.400	0.401–0.471	0.400–0.584	0.413–0.569	0.428–0.875
*γ*_*Sb*3_ (/s)	0.400	0.401–0.473	0.414–0.593	0.466–0.721	0.412–0.853
*K*_*I*_	0.045	0.048–0.054	0.048–0.061	0.052–0.086	0.058–0.085
*K*_*P*_	1.000	1.016–1.165	1.130–1.359	1.137–1.549	1.159–1.367
*γ*_*K*1_ (/s) [10 ^−3^]	0.400	0.436–0.477	0.424–0.515	0.434–0.674	0.505–0.795
*γ*_*K*2_ (/s) [10 ^−3^]	0.400	0.403–0.466	0.401–0.454	0.473–0.666	0.570–0.683
*γ*_*Sm*1_ (/s)	0.800	0.809–0.948	0.863–1.099	0.827–1.544	0.825–1.410
*γ*_*Sm*2_ (/s)	0.800	0.835–0.943	0.904–1.012	0.849–1.205	1.152–1.548
*γ*_*Sm*3_ (/s)	0.800	0.832–0.958	0.823–1.140	0.841–1.536	0.853–1.279
*k*_1_ (/s)	0.100	0.101–0.116	0.106–0.142	0.111–0.174	0.127–0.208
*k*_2_ (/s)	0.100	0.101–0.114	0.102–0.139	0.103–0.199	0.123–0.211
CMC controller					
Characteristics	Nominal	*Δ*=0.2	*Δ*=0.5	*Δ*=1.0	*Δ*=1.2
*t*_*r*_ (s)	29	37	65	89	117
*t*_*s*_ (s)	97	116	155	202	353
*M*_*OV*_ (%)	10.12	25.3	44.63	60.55	75.00
*e*_*ss*_ (M)	0.00	0.18	0.46	0.92	1.12
Parameters	Nominal	*Δ*=0.2	*Δ*=0.5	*Δ*=1.0	*Δ*=1.2
*γ*_*Sb*1_ (/s)	0.400	0.403–0.476	0.450–0.595	0.412–0.781	0.626–0.857
*γ*_*Sb*2_ (/s)	0.400	0.401–0.477	0.406–0.580	0.407–0.752	0.403–0.745
*γ*_*Sb*3_ (/s)	0.400	0.403–0.466	0.402–0.594	0.577–0.726	0.425–0.806
*k*_*b*1_ (/M/s) [10 ^−2^]	0.185	0.186–0.221	0.202–0.274	0.199–0.342	0.224–0.365
*k*_*b*2_ (/s)	0.500	0.514–0.565	0.516–0.683	0.621–0.737	0.509–0.747
*k*_*b*3_ (/M/s) [10 ^−2^]	0.185	0.187–0.213	0.187–0.245	0.242–0.354	0.230–0.318
*k*_*b*4_ (/s)	0.500	0.516–0.596	0.679–0.723	0.553–0.851	0.662–1.008
*k*_1_ (/s)	0.100	0.100–0.119	0.104–0.148	0.130–0.199	0.101–0.174
*k*_2_ (/s)	0.100	0.101–0.114	0.105–0.150	0.106–0.198	0.109–0.209

The performance of the two nominal closed-loop systems is rather similar, which reflects the fact that the CMC controller is designed to reproduce the steady-state input-output mapping of the original PI controller. Interestingly, however, we can clearly see a significantly improved robustness of the system when the CMC controller is used. With the PI controller, the closed-loop system become unstable when *Δ*=1.2, while for the CMC controller, the closed-loop system becomes unstable only when *Δ*=1.8, showing that the CMC controller is able to tolerate more than a 50 % larger variability in the values of the reaction rates in the underlying chemical reactions.

#### Performance analysis of controllers with nonlinear process

We proceed to analyse how the two controllers fare in controlling a more complex second-order nonlinear process. The same step test and Monte Carlo simulations are carried out, with the parameters for the nominal system in the required abstract chemical reactions given as:

*Nonlinear process:* *k*_*r*1_=0.00005 /M/s, *k*_*r*2_=1.6 /s, *k*_*r*3_=0.0008 /s, with the total concentration constrained so that *p*+*q*=5.5 M.

*PI controller:* *γ*_*Sb*1_, *γ*_*Sb*2_, *γ*_*Sb*3_, *γ*_*Sm*1_, *γ*_*Sm*2_, *γ*_*Sm*3_, *γ*_*K*1_, *γ*_*K*2_=0.0004 /s, *K*_*P*_=0.65 and *K*_*I*_=0.3.

*CMC controller:* *k*_1_==0.0000055 /M/s, *k*_3_=0.000018 /M/s, *k*_2_=12.50 /s, *k*_4_=140 /s, *x*_*p*_+*u*+*x*_*C*1_+*x*_*C*2_=66 M and *x*_*e*_+*x*_*C*2_=0.00012 M.

*Subtractor dynamics:* *γ*_*Sb*1_, *γ*_*Sb*2_, *γ*_*Sb*3_=0.4 /s.

**Fig. 7:**
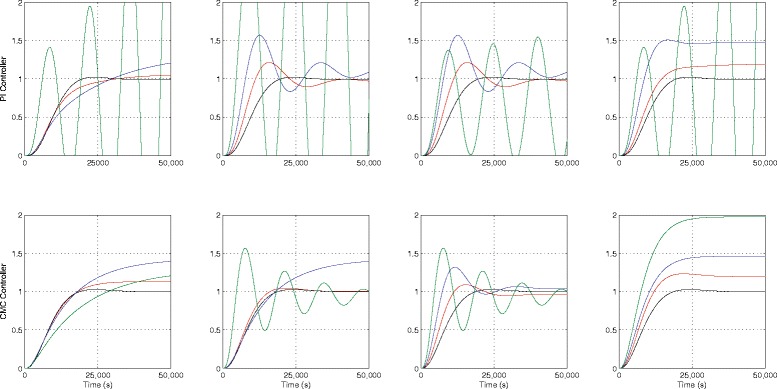
*Top* row: PI controller + nonlinear process. *Bottom row*: CMC controller + nonlinear process. Nominal and worst cases of *t*_*r*_, *t*_*s*_, *M*_*OV*_ and *e*_*ss*_ with *Δ* = 0.2, 0.5 and 1.0. *Black* line: Nominal system. *Red* line: worst-case response for *Δ* = 0.2, *Blue* line: worst-case response for *Δ* = 0.5, *Green* line: worst-case response for *Δ* = 1.0

The step response characteristics for both the nominal systems are tabulated in Table 2. As previously, the step responses depicting the nominal and worst-case responses for *Δ*∈{0.2,0.5,1.0} are shown in Fig. 7 for the PI and CMC controllers respectively. Note that we do not consider the case for *Δ*=1.2 as the closed-loop system becomes unstable for *Δ*=1.0, when the PI controller + nonlinear process is used.

**Table 2:** Step response characteristics and worst-case parameter ranges for PI and CMC controllers + nonlinear process

PI controller				
Characteristics	Nominal	*Δ*=0.2	*Δ*=0.5	*Δ*=1.0
*t*_*r*_ (s)	11,139	18,959	31,673	Unstable
*t*_*s*_ (s)	26,304	44,138	48,482	Unstable
*M*_*OV*_ (%)	2.42	24.88	44.44	Unstable
*e*_*ss*_ (M)	0.00	0.19	0.48	Unstable
Parameters	Nominal	*Δ*=0.2	*Δ*=0.5	*Δ*=1.0
*γ*_*Sb*1_ (/s) [10 ^−3^]	0.400	0.445-0.480	0.492-0.596	0.472-0.730
*γ*_*Sb*2_ (/s) [10 ^−3^]	0.400	0.402-0.475	0.400-0.453	0.533-0.735
*γ*_*Sb*3_ (/s) [10 ^−3^]	0.400	0.427-0.467	0.433-0.591	0.402-0.473
*K*_*I*_ [10 ^−3^]	0.300	0.301-0.353	0.310-0.420	0.486-0.504
*K*_*P*_	0.650	0.673-0.771	0.790-0.908	0.806-1.282
*γ*_*K*1_ (/s) [10 ^−3^]	0.400	0.432-0.461	0.446-0.573	0.498-0.747
*γ*_*K*2_ (/s) [10 ^−3^]	0.400	0.401-0.422	0.446-0.586	0.404-0.740
*γ*_*Sm*1_ (/s) [10 ^−3^]	0.400	0.427-0.479	0.424-0.587	0.641-0.791
*γ*_*Sm*2_ (/s) [10 ^−3^]	0.400	0.423-0.462	0.469-0.553	0.615-0.730
*γ*_*Sm*3_ (/s) [10 ^−3^]	0.400	0.409-0.478	0.418-0.539	0.401-0.431
*k*_*r*1_ (/M/s) [10 ^−4^]	0.500	0.509-0.594	0.536-0.734	0.737-0.945
*k*_*r*2_ (/s)	1.600	1.633-1.865	1.749-2.232	1.860-2.843
*k*_*r*2_ (/s) [10 ^−3^]	0.800	0.812-0.904	0.819-1.102	0.844-0.891
CMC controller				
Characteristics	Nominal	*Δ*=0.2	*Δ*=0.5	*Δ*=1.0
*t*_*r*_ (s)	11,147	15,501	25,753	30,838
*t*_*s*_ (s)	28,848	28,324	42,494	49,196
*M*_*OV*_ (%)	2.84	13.12	26.25	56.37
*e*_*ss*_ (M)	0.00	0.19	0.46	0.98
Parameters	Nominal	*Δ*=0.2	*Δ*=0.5	*Δ*=1.0
*γ*_*Sb*1_ (/s) [10 ^−3^]	0.400	0.426-0.479	0.534-0.597	0.542-0.798
*γ*_*Sb*2_ (/s) [10 ^−3^]	0.400	0.400-0.478	0.404-0.511	0.403-0.798
*γ*_*Sb*3_ (/s) [10 ^−3^]	0.400	0.406-0.457	0.425-0.578	0.403-0.633
*k*_*b*1_ (/M/s) [10 ^−5^]	0.550	0.567-0.644	0.577-0.808	0.619-1.075
*k*_*b*2_ (/s)	12.50	12.64-14.75	16.57-17.84	16.99-17.95
*k*_*b*3_ (/M/s) [10 ^−4^]	0.180	0.182-0.207	0.203-0.238	0.196-0.274
*k*_*b*4_ (/s)	140.00	144.48-163.09	165.38-205.25	143.15-278.30
*k*_*r*1_ (/M/s) [10 ^−4^]	0.500	0.503-0.593	0.523-0.712	0.538-0.807
*k*_*r*2_ (/s)	1.600	1.635-1.893	1.839-1.950	1.789-2.861
*k*_*r*2_ (/s) [10 ^−3^]	0.800	0.803-0.943	0.804-1.162	0.808-1.525

The performance of the two nominal closed-loop systems are rather similar, which again reflects the fact that the CMC controller is designed to reproduce the steady-state input-output mapping of the original PI controller. The closed-loop system with the CMC controller retains closed-loop stability up until *Δ*=1.6, again demonstrating a significantly higher level of robustness than exhibited by the linear PI controller.

#### Flexible input-output mapping improves robustness

**Fig. 8:**
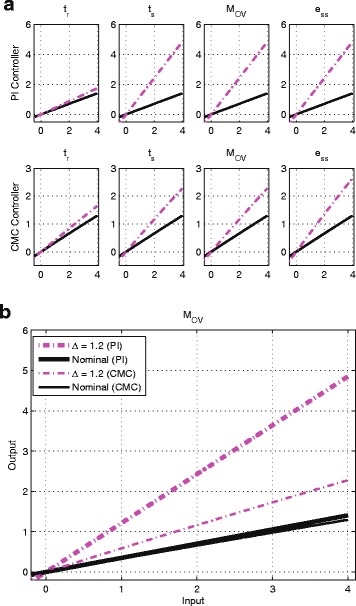
**a** The mapping of steady-state input-output signals of the PI controller (*top* row) and the CMC controller (*bottom* row) when controlling the linear process. Black solid line: Nominal system. Magenta dash-dotted line: worst-case response for *Δ* = 1.2. **b** The zoomed-in version of *M*_*OV*_ from (A). *Black* solid line: nominal system. *Magenta* dash-dotted line: worst-case response for *Δ* = 1.2

The results thus far have shown consistently better robustness from the CMC controller compared to the PI controller. To explain this, we analyse the mapping of steady-state input-output signals of these two controllers. Fig. 8(a) shows the mapping of steady-state input-output signals of both controllers as they were implemented when controlling the linear process. The mapping of input-output signals for the nominal system and the maximum deviation from this response when *Δ*=1.2 are shown in black solid line and magenta dash-dotted line respectively. We observe a significantly greater change to the gradient of the PI controller’s input-output mappings compared to the CMC controller.

This intriguing observation leads us to ask how the gradient of this mapping of steady-state input-output signals is related to the robustness of the controller? Here, the gradient is associated with the straight line equation, *y*=*mx*+*c*, where *m* is the gradient and *c* is the intersection of the y-axis given that the mapping of the steady-state input-output signals are made up of a straight line.

Given the process to be controlled is a linear process, its ODE representation (with *x*:=*y*) is given by

12 $$  \frac{dx}{dt} = -k_{p2}x + k_{p1}u  $$

Equation 12 is in the standard state-space representation (i.e. $\frac {dx}{dt} = Ax + Bu, y = Cx + Du$) with *A*=−*k*_*p*2_ and *B*=*k*_*p*1_, *C*=1 and *D*=0. In linear control theory design using a state-space approach, [42], a standard control law can be written as *u*=*Kx* where *K* is the controller gain. This linear control law can be viewed as a mapping of input, *x* to output, *u* with *K* being the gradient. Substituting *u*=*Kx* into Eq. (12), we have

13 $$\begin{array}{*{20}l} \frac{dx}{dt} & = -k_{p2}x + k_{p1}Kx = \left(k_{p1}K - k_{p2}\right)x \end{array} $$

As Eq. (13) is in scalar form, the overall process is stable if the real part of the eigenvalue of *A* (i.e. *k*_*p*1_ *K*−*k*_*p*2_) is less than 0, hence, the following condition, $K < \frac {k_{p2}}{k_{p1}}$ must hold. In other words, if the controller gain, *K* is less than the ratio of the process parameters *k*_*p*2_ to *k*_*p*1_, we have a stable system. In our simulation, the process parameters of the nominal system are *k*_*p*1_,*k*_*p*2_=0.1, thus, for the system to be stable, we require $K < \frac {k_{p2}}{k_{p1}} = 1$. Looking at Fig. 8(b), a zoomed-in version using *M*_*OV*_ as illustration, the gradient of both the controllers’ input-output mapping are less than 1 (i.e. ≈0.34); the closed-loop system is stable. Note that for the nominal system, both the controllers’ input-output mappings are very similar, as expected, since the CMC controller was designed to reproduce the PI controller’s steady-state input-output mapping.

We now consider the effect of increasing levels of variability in the values of the parameters in the chemical reactions implementing the feedback control system. For the PI controller, at *Δ*=1.2, the process parameters change from *k*_*p*1_=0.100→0.208 and *k*_*p*2_=0.100→0.124. Thus, the ratio $\frac {k_{p2}}{k_{p1}}$ changes from 1 →0.596. Likewise, from Fig. 8(b), we see the gradient of the PI controller’s steady-state input-output mapping changes to 1.213 $> \frac {k_{p2}}{k_{p1}} = 0.596$, which accounts for the observed unstable behaviour.

On the other hand, the change of gradient for the CMC controller is smaller compared to the PI controller. At *Δ*=1.2, the process parameters change from *k*_*p*1_=0.1→0.174 and *k*_*p*2_=0.1→0.109, leading the ratio $\frac {k_{p2}}{k_{p1}} $ to change from 1 →0.628. However, the gradient of the CMC controller’s steady-state input-output mapping changes to 0.588 $< \frac {k_{p2}}{k_{p1}} = 0.628$, thus preserving the stability of the system.

**Fig. 9:**
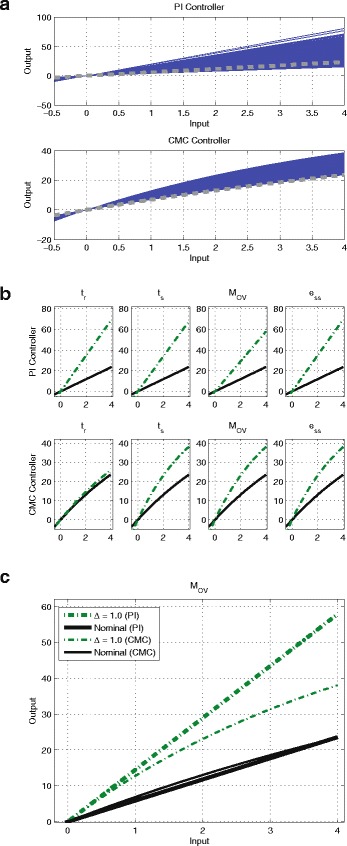
**a** The mappings of steady-state input-output signals of PI controller and CMC controller simulated 1060 times for *Δ* = 1.0. Nominal systems are shown in thick dotted grey line. **b** The mapping of steady-state input-output signals of the PI controller (*top* row) and the CMC controller (*bottom* row) when controlling the nonlinear process. *Black* solid line: Nominal system. *Green* dash-dotted line: worst-case response for *Δ* = 1.0. **c** The zoomed-in version of the mapping of steady-state input-output signals of the PI controller (bold line) and the CMC controller (thin line) for *M*_*OV*_ from (B). *Black* solid line: Nominal system. *Green* dash-dotted line: worst-case response for *Δ* = 1.0

What makes the CMC more robust (in terms of gradient change) to parameter uncertainty? Our simulation results using the nonlinear process shed some light on this matter. The steady-state mapping of input-output signals simulated 1060 times at *Δ*=1.0 for both the controllers when controlling the nonlinear process is shown in Fig. 9(a). For the nominal system both the controllers’ input-output mapping retains a the linear behaviour. While the PI controller’s steady-state input-output mapping stays linear for all 1060 uncertainty combinations, the CMC controller’s input-output mapping displays a ‘hyperbolic’ behaviour for some parameter combinations. Recall that this ‘hyperbolic’ behaviour is one of the input-output signal mappings reported in [25] (see also Fig. 5(b)). Thus, our simulation results seem to indicate that parameter uncertainty has the capacity to change the operating regime of the CMC controller from signal-transducing to hyperbolic. Thus, the question we are interested in is whether this change in the mapping regime accounts for the better robustness of the CMC controller.

As the process is now nonlinear, the notion of eigenvalue no longer applies while the notion of stability for a nonlinear system is also more mathematically involved and beyond the scope of this paper. However, we can informally explain the difference in the robustness of both controllers by extending our arguments on the ‘gradient’ of the steady-state input-output mapping, as was done for the linear process. Figure 9(b) shows the nominal and worst-case deviation in the input-output mapping for both controllers at *Δ*=1.0.

From Fig. 9(c), we can see that despite both controllers having very similar mapping of input-signal signals for the nominal system, when subjected to parameter uncertainty, the gradient of the PI controller’s steady-state input-output mapping becomes steeper and subsequently affects the stability of the system. On the other hand, not only does the CMC controller’s input-output mapping show a smaller change in response to uncertainty, it becomes more hyperbolic. The CMC controller’s innate ability to achieve hyperbolic behaviour seems to be able to prevent the adverse effect of parameter uncertainty, as it enables the gradient of its input-output mapping when subjected to parameter uncertainty to remain small. In this particular case, we show through an extensive analysis that the change in the mapping behaviour of the CMC controller from the linear signal-transducing regime to the hyperbolic regime actually acts to improve the robustness of the CMC controller.

## Conclusions

In this paper, we have shown how the set of chemical reactions underlying the covalent modification cycle motif may be used to design a range of analog biomolecular circuits for computation, information processing and control. By exploiting the four distinct input-output mapping behaviours of the covalent modification cycle, we have designed biomolecular circuits to compute complex nonlinear operators and implement nonlinear feedback controllers. Our design approach results in a dramatic reduction in circuit complexity compared to the use of purely abstract reactions from standard chemical reaction network theory, and requires far fewer chemical reactions to be implemented experimentally. Our designs also demonstrated significantly greater robustness to variability in circuit parameters that will inevitably arise in experimental implementations of synthetic circuitry. Given the range of input-output mappings that can be produced by the set of chemical reactions underlying the covalent modification cycle, it is likely that they could be used to efficiently design many other types of operators and controllers. As the chemical reactions concerned are all represented either in unimolecular or bimolecular form, the resulting circuits can then be readily implemented using DNA-based chemistry either in vitro or in vivo for future Synthetic Biology applications.

## Abbreviations

DNA: Deoxyribonucleic acid
DSD: DNA strand displacement
ODE: Ordinary differential equation
PI: proportional-integral
CMC: Covalent modification cycle
